# Supplementary figures and images for: S6K1 phosphorylates Cdk1 and MSH6 to regulate DNA repair
Source: eLife. 2022 Oct 3;11:e79128. doi: 10.7554/eLife.79128 (PMC9529248; doi:10.7554/eLife.79128)

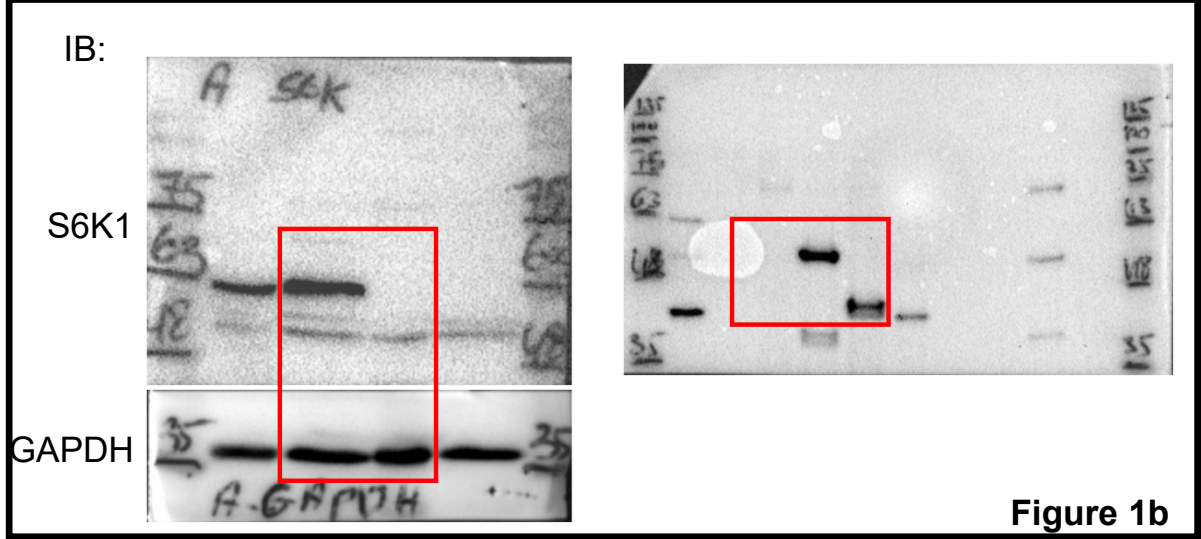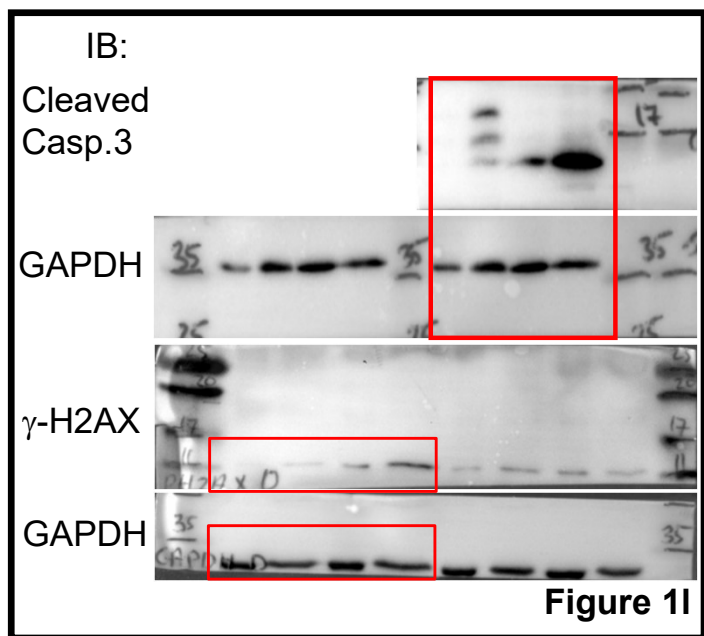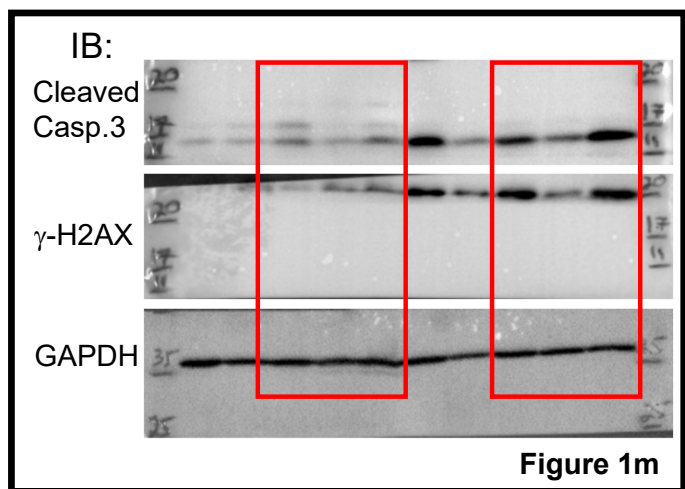

Supplement: Figure 1—source data 1. [file elife-79128-fig1-data1.pdf]

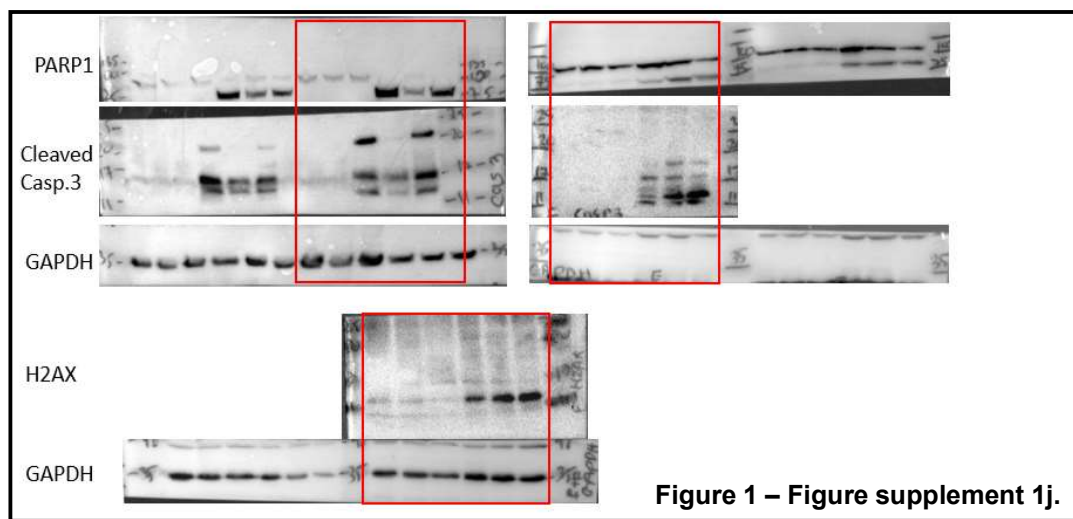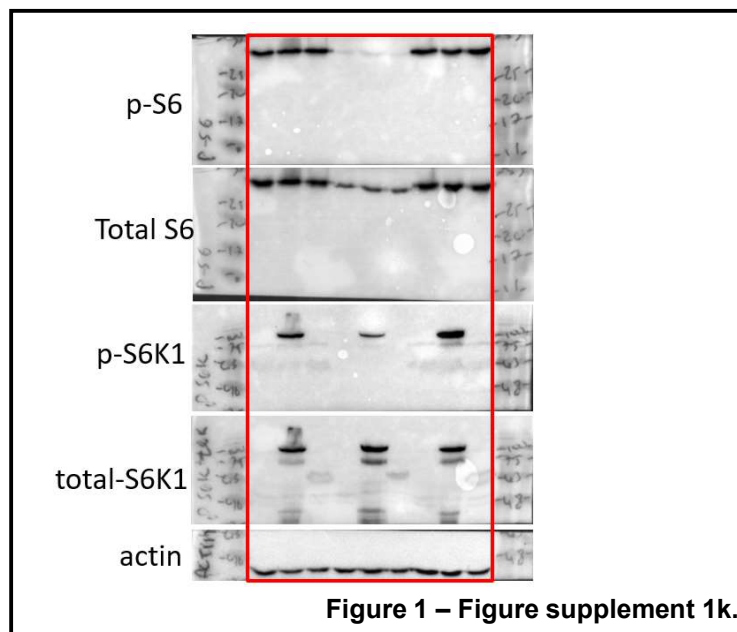

Supplement: Figure 1—figure supplement 1—source data 1. [file elife-79128-fig1-figsupp1-data1.pdf]

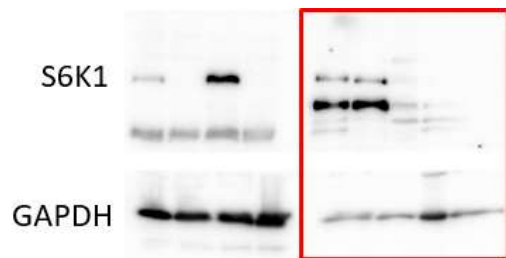

Figure 1 – Figure supplement 2c.

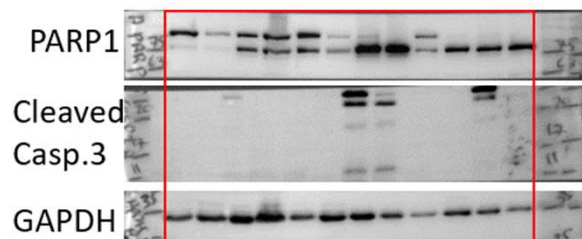

Figure 1 – Figure supplement 2d.

Supplement: Figure 1—figure supplement 2—source data 1. [file elife-79128-fig1-figsupp2-data1.pdf]

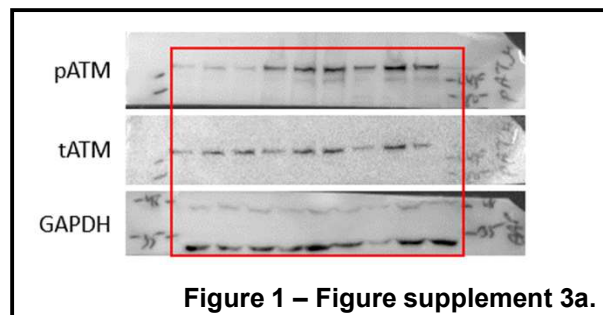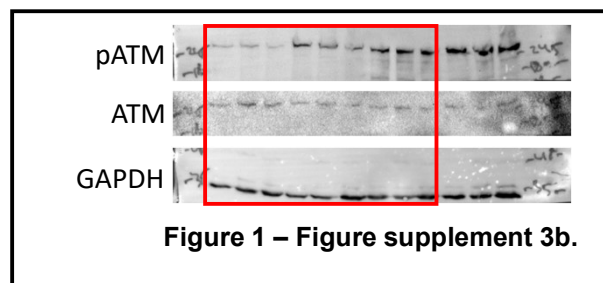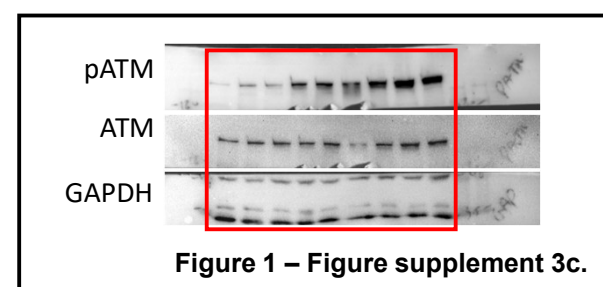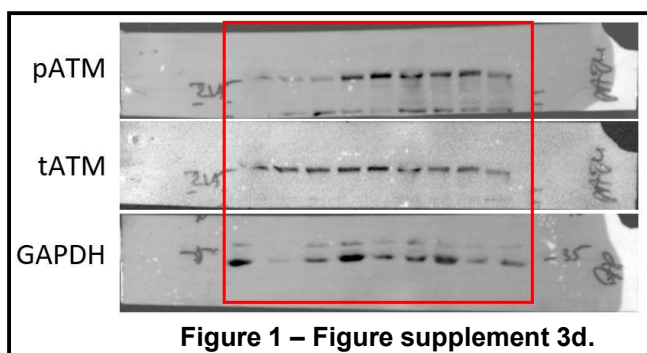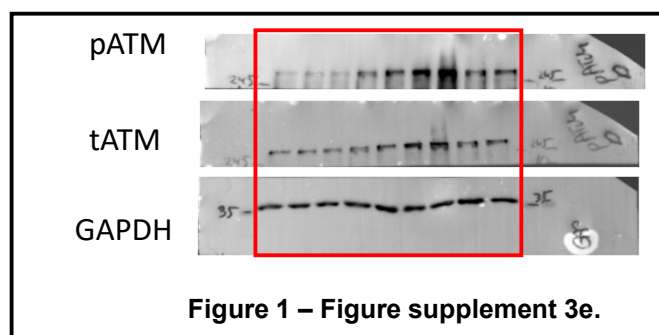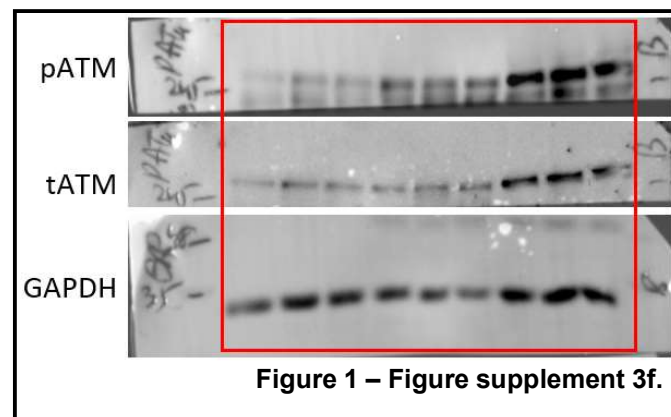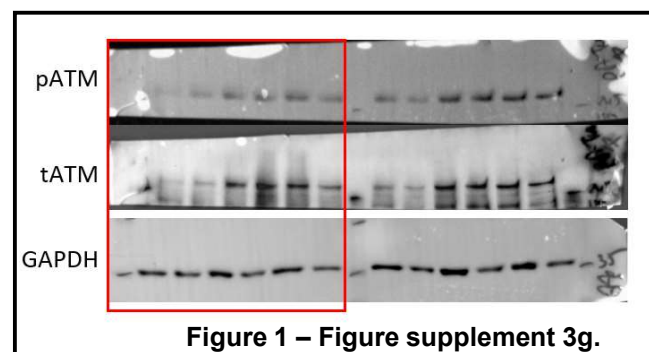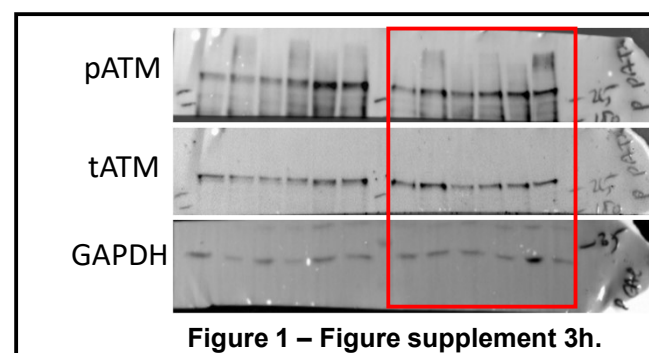

Supplement: Figure 1—figure supplement 3—source data 1. [file elife-79128-fig1-figsupp3-data1.pdf]

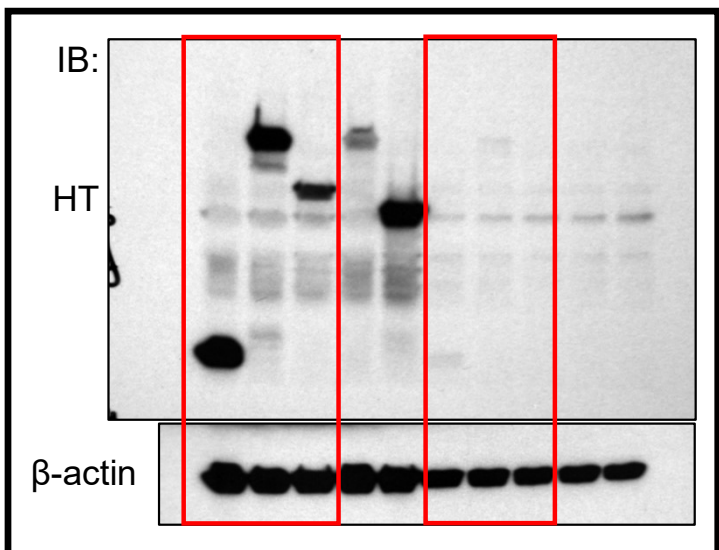

Figure 2d

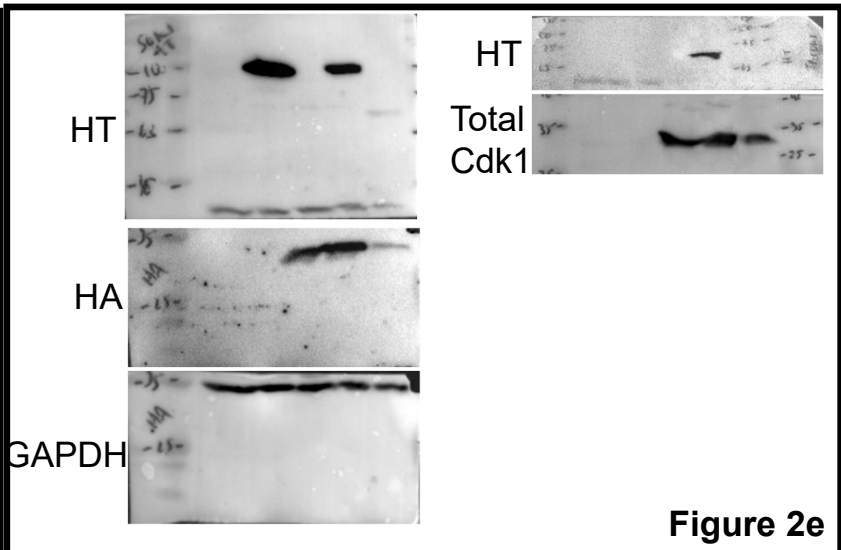

Figure 2e

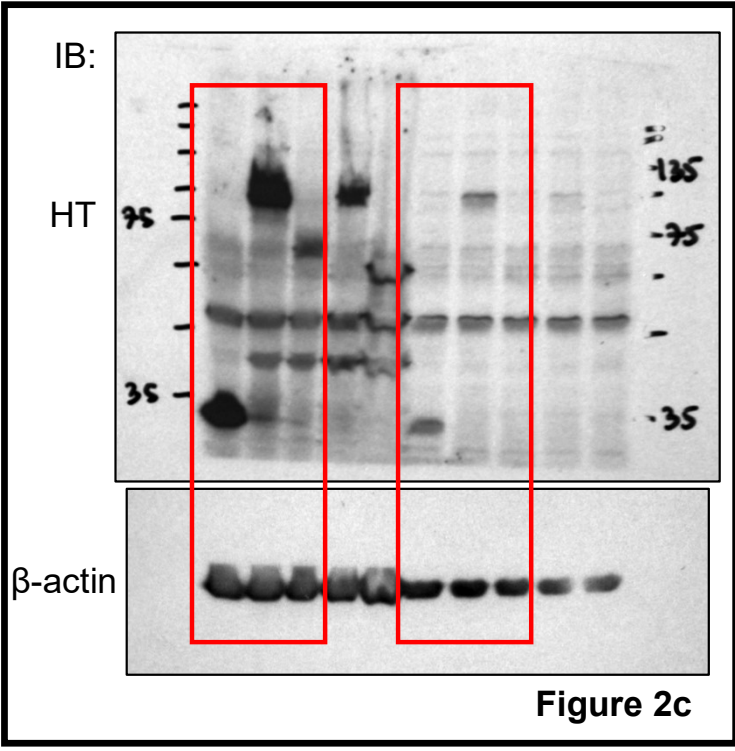

Figure 2c

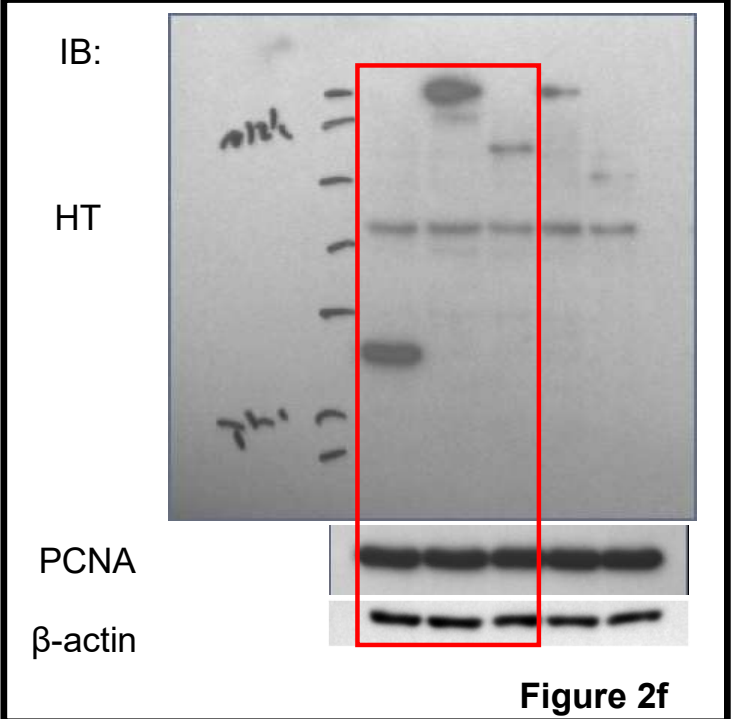

Figure 2f

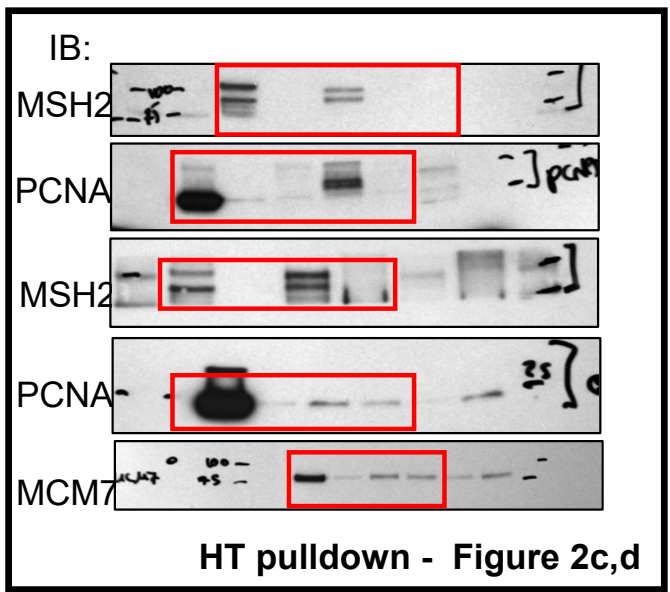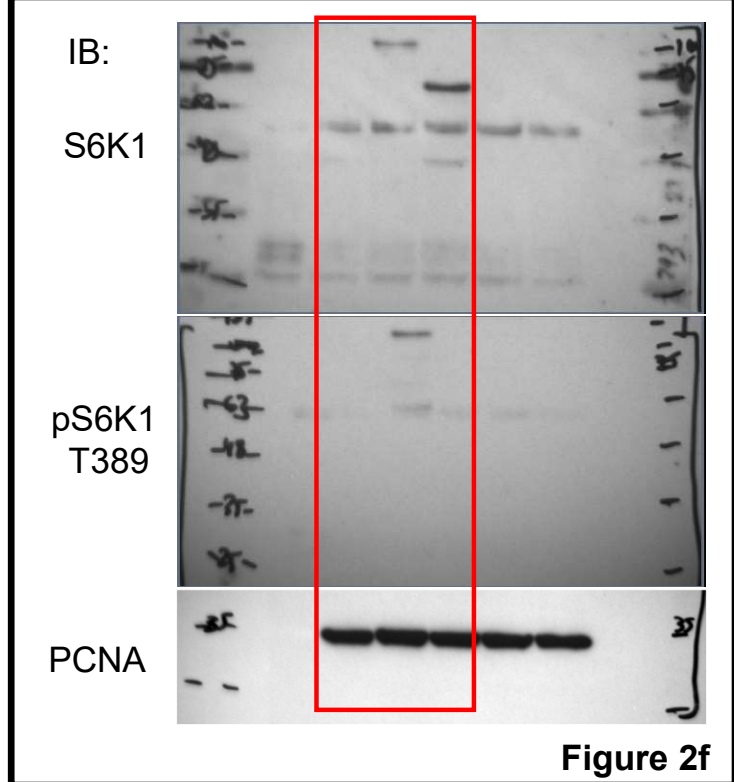

Figure 2f

Supplement: Figure 2—source data 1. [file elife-79128-fig2-data1.pdf]

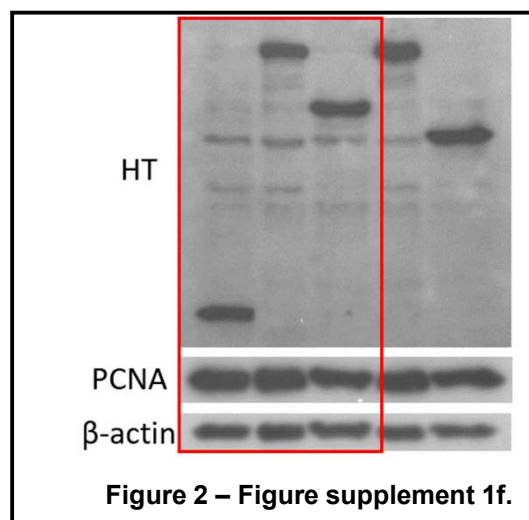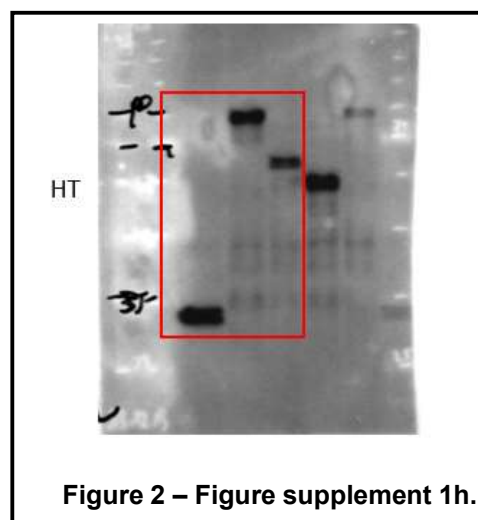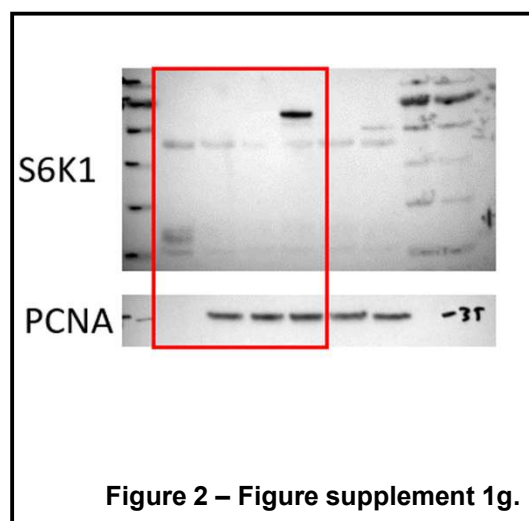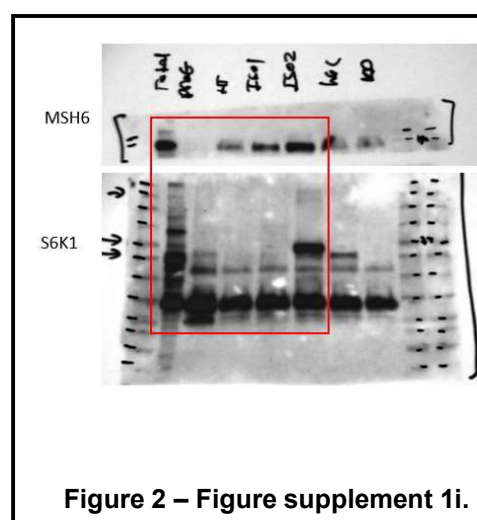

Supplement: Figure 2—figure supplement 1—source data 1. [file elife-79128-fig2-figsupp1-data1.pdf]

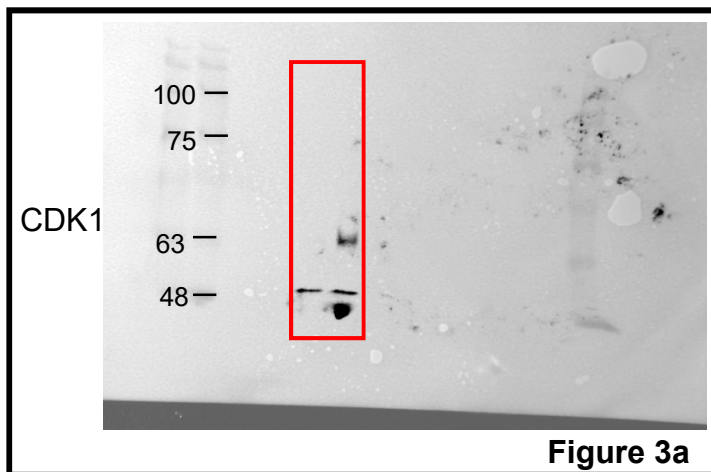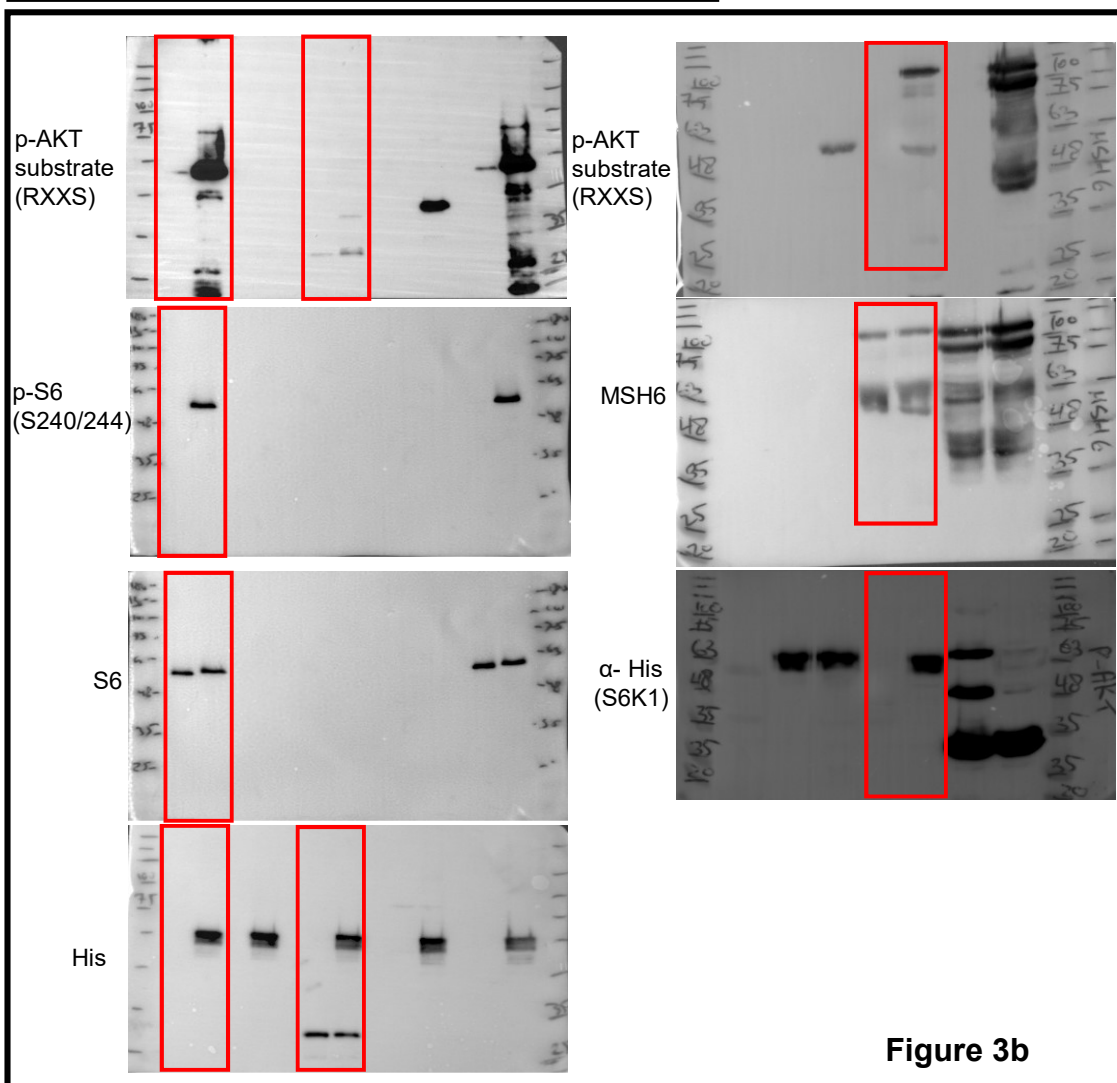

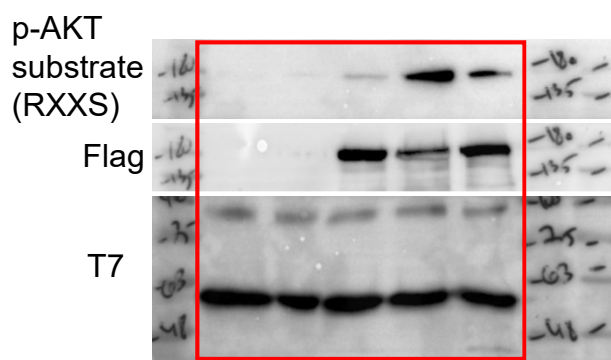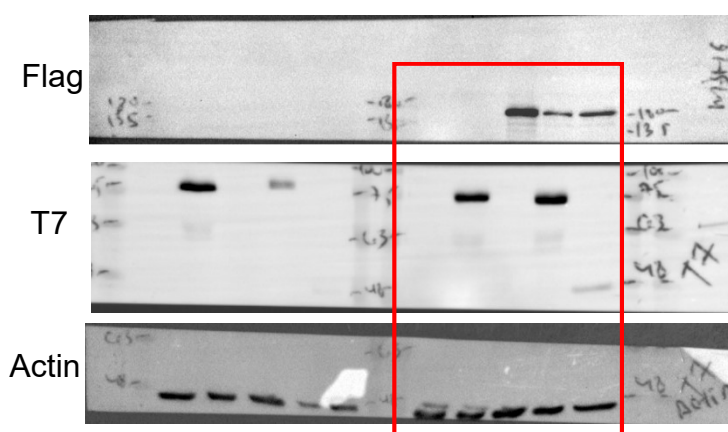

**Figure 3c**

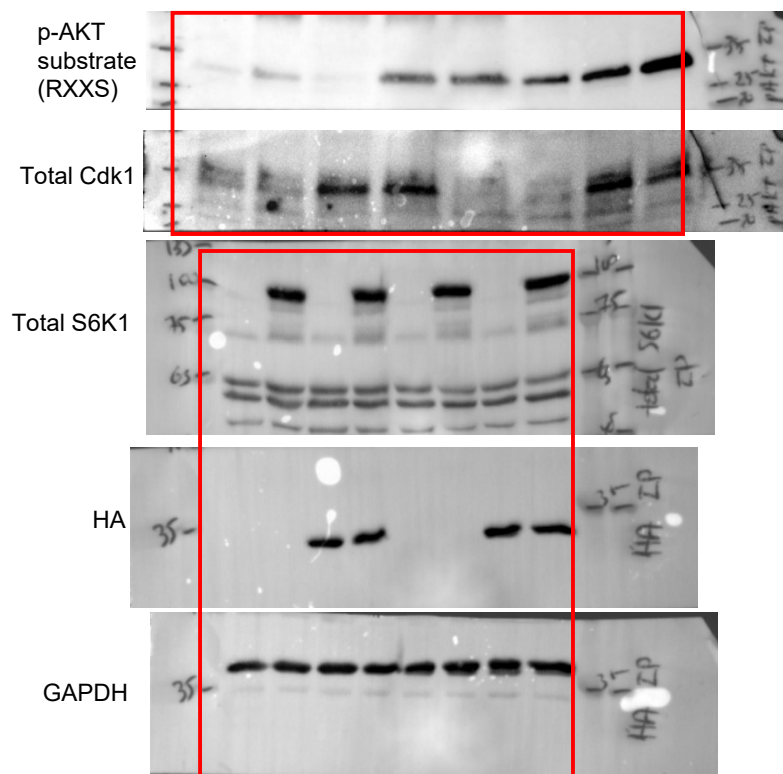

**Figure 3e**

Supplement: Figure 3—source data 1. [file elife-79128-fig3-data1.pdf]

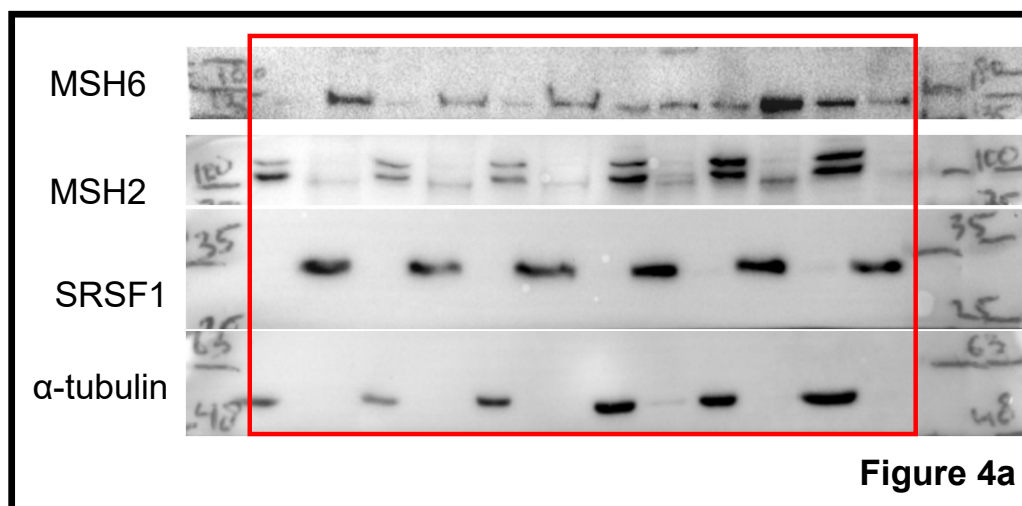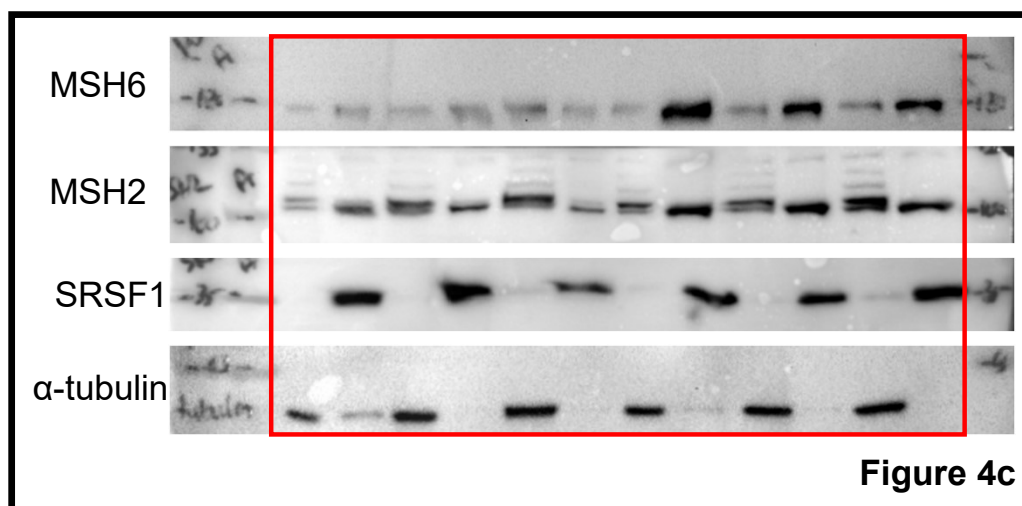

Supplement: Figure 4—source data 1. [file elife-79128-fig4-data1.pdf]

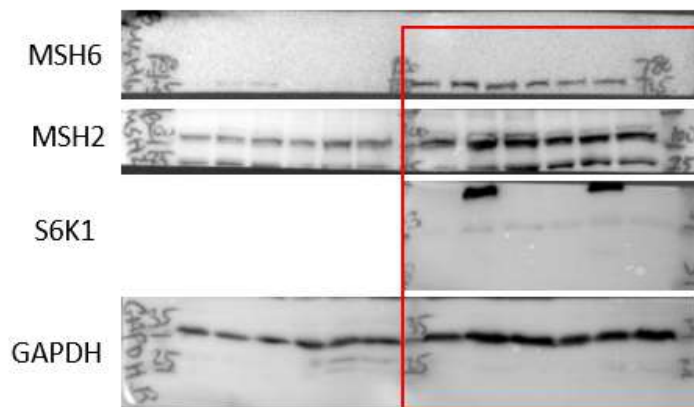

Figure 4 – Figure supplement 1a.

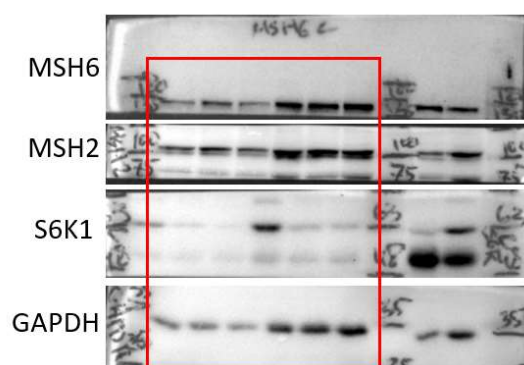

Figure 4 – Figure supplement 1b.

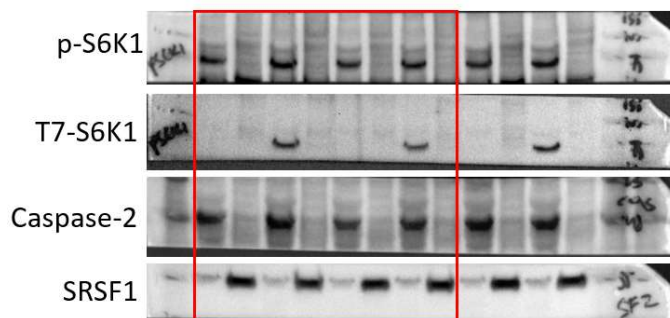

Figure 4 – Figure supplement 1c.

Supplement: Figure 4—figure supplement 1—source data 1. [file elife-79128-fig4-figsupp1-data1.pdf]
