# Supplementary material for: S6K1 phosphorylates Cdk1 and MSH6 to regulate DNA repair
Source: eLife. 2022 Oct 3;11:e79128. doi: 10.7554/eLife.79128 (PMC9529248; doi:10.7554/eLife.79128)
Supplement: Figure 6—source data 1. [file elife-79128-fig6-data1.pdf]

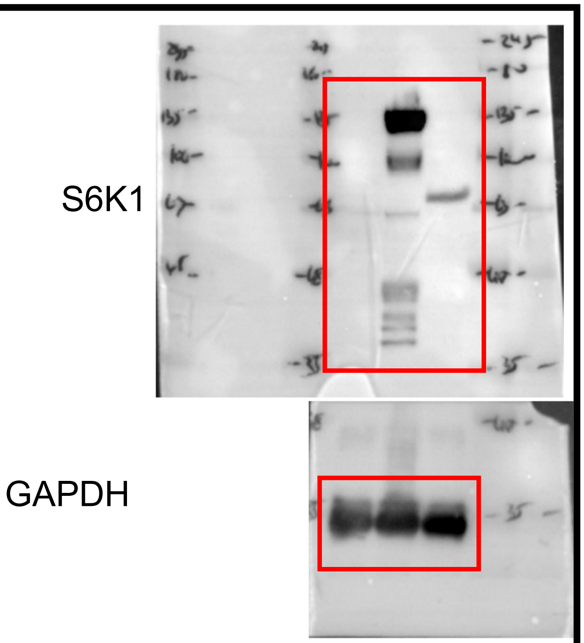

Figure 6b

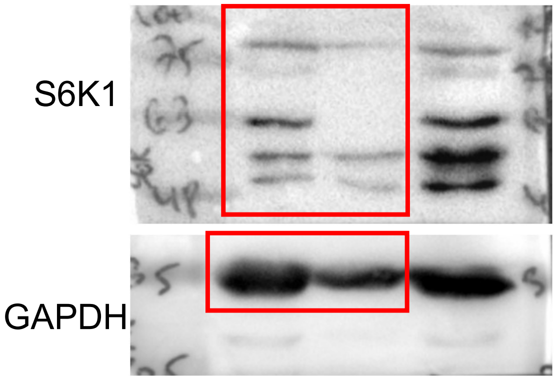

Figure 6c

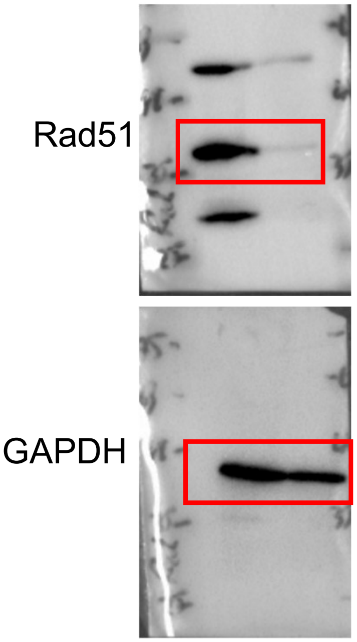

Figure 6d

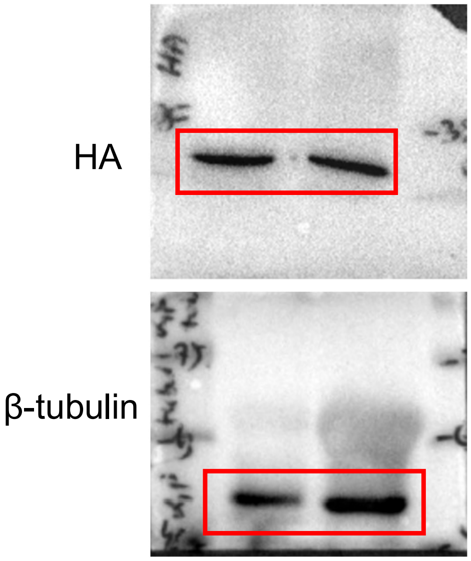

Figure 6e

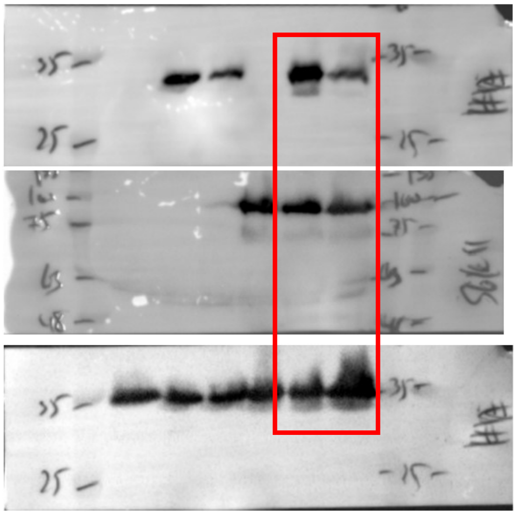
Figure 6f

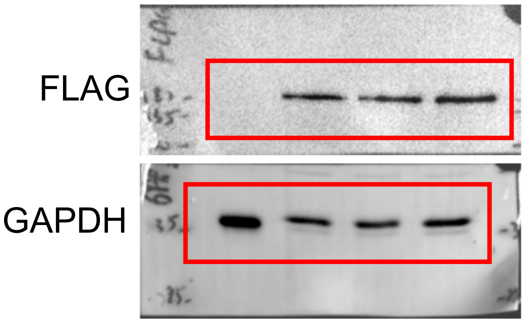

Figure 6i
